# Evaluating primary suicide prevention in adolescents with risk factors (ESPAIR): study protocol for a cluster-randomized controlled trial

**DOI:** 10.1186/s13063-025-09266-y

**Published:** 2025-11-24

**Authors:** Stéphanie Baggio, Eleanor Bailey, Anne Edan, Patrick Heller, Carole Kapp, Nadejda Lambert, Laurent Michaud, Adrian P. Mundt, Neslie Nsingi, Sophia Perez, Santiago Peregalli, Camille Piguet, Caroline Piotrowski, Yekaterina Prutyanova, Mathias Rumley, Marlène Sapin, Sébastien Urben, Katia Iglesias

**Affiliations:** 1 Institute of Psychology, University of Lausanne, Lausanne, Switzerland; 2 Institute of Primary Health Care (BIHAM), University of Bern, Bern, Switzerland; 3 Orygen Centre for Youth Mental Health, University of Melbourne, Melbourne, Australia; 4 Child and Adolescent Psychiatric Clinical Service, Geneva University Hospitals, Geneva, Switzerland; 5 Psychiatry Department, Geneva University Hospitals, Geneva, Switzerland; 6 Division of Prison Health, Geneva University Hospitals, Geneva, Switzerland; 7 Division of Child and Adolescent Psychiatry, Department of Psychiatry, Lausanne University Hospital and University of Lausanne, Lausanne, Switzerland; 8 Association Stop Suicide, Geneva, Switzerland; 9 Psychiatric Liaison Service, Lausanne University Hospital, Lausanne, Switzerland; 10 Centro de Investigación Biomédica, Facultad de Medicina, Universidad Diego Portales, Santiago, Chile; 11 Department of Psychiatry and Mental Health, Facultad de Medicina Norte, University of Chile, Santiago, Chile; 12 College of Community & Global Health, University of Manitoba, Winnipeg, Canada; 13 Psychiatry Department, Faculty of Medicine, University of Geneva, Geneva, Switzerland; 14 Primary and Secondary School Center Lavaux, Puidoux, Switzerland; 15 Swiss Center of Expertise in Social Sciences (FORS) & Interdisciplinary Center of Life Course Research (LIVES), University of Lausanne, Lausanne, Switzerland; 16 School of Health Sciences, HES-SO University of Applied Sciences and Arts of Western Switzerland, Fribourg, Switzerland

**Keywords:** Universal prevention, Psychoeducation, Mental health, LGBTQIA+, Prison, School disengagement, Chronic condition, Foster care

## Abstract

**Background:**

Suicide is the leading cause of death among young people. Because it is preventable, suicide prevention has become a critical public health priority worldwide. Current evidence suggests that primary suicide prevention interventions are effective in promoting suicide awareness and reducing suicidal thoughts among adolescents. However, scarce research has been conducted in vulnerable populations who may be at increased risk of suicidal behaviour. This project aims to test the effectiveness of a primary suicide prevention intervention on suicide awareness, knowledge of suicide and local resources, suicidal thoughts, psychological distress, and safety.

**Methods:**

This study is an open-label, multi-centre, superiority, cluster-randomized controlled trial with two parallel arms (randomization 1:1 in at least 8 study sites) of a primary suicide prevention intervention based on psychoeducation versus an art-based control intervention. Four groups of youth aged 14–25 with high suicide risk will be recruited (in total, *n* = 240), including those (1) detained in juvenile detention centres, (2) disconnected from school, (3) in treatment for psychiatric disorders, (4) belonging to a sexual or gender diversity, (5) affected by chronic conditions, and (6) with relational/familial problems. Participants will be randomly assigned to the suicide prevention or control intervention. The primary outcome is suicide awareness measured on a validated scale. Secondary outcomes include knowledge of suicide, knowledge of local resources, suicidal behaviour, psychological distress, access to primary suicide prevention, and safety. Measures will be collected at baseline, at 1-week, and 3-month follow-ups. Analyses will be conducted as intention-to-treat using mixed-effects models.

**Discussion:**

We expect that a brief primary suicide prevention intervention (psychoeducation) will be effective and safe in vulnerable adolescents.

**Trial registration:**

ClinicalTrials.gov NCT06551038. Registered on August 13, 2024.

**Supplementary Information:**

The online version contains supplementary material available at 10.1186/s13063-025-09266-y.

## Administrative information

**Table:** 

Title {1}	Evaluating primary suicide prevention in adolescents with risk factors (ESPAIR): study protocol for a cluster-randomized controlled trial
Trial registration {2a and 2b}	ClinicalTrials.gov ID NCT06551038
Protocol version {3}	Version 6, August 19, 2025
Funding {4}	This study was supported by the Swiss National Science Foundation (grant no. 10000272)
Author details {5a}	^1^Institute of Psychology, University of Lausanne, Lausanne, Switzerland ^2^Institute of Primary Health Care (BIHAM), University of Bern, Bern, Switzerland ^3^Orygen Centre for Youth Mental Health, University of Melbourne, Melbourne, Australia ^4^Child and Adolescent Psychiatric Clinical Service, Geneva University Hospitals, Geneva, Switzerland ^5^Psychiatry Department, Geneva University Hospitals, Geneva, Switzerland ^6^Division of Prison Health, Geneva University Hospitals, Geneva, Switzerland ^7^Division of Child and Adolescent Psychiatry, Department of Psychiatry, Lausanne University Hospital and University of Lausanne, Lausanne, Switzerland ^8^Association Stop Suicide, Geneva, Switzerland ^9^Psychiatric Liaison Service, Lausanne University Hospital, Lausanne, Switzerland ^10^Centro de Investigación Biomédica, Facultad de Medicina, Universidad Diego Portales, Santiago, Chile ^11^Department of Psychiatry and Mental Health, Facultad de Medicina Norte, University of Chile, Santiago, Chile ^12^College of Community & Global Health, University of Manitoba, Winnipeg, Canada ^13^Psychiatry Department, Faculty of Medicine, University of Geneva, Geneva, Switzerland ^14^Primary and Secondary School Center Lavaux, Puidoux, Switzerland ^15^Swiss Center of Expertise in Social Sciences (FORS) and Interdisciplinary Centre for Life Course Research (LIVES), University of Lausanne, Lausanne, Switzerland ^16^School of Health Sciences, HES-SO University of Applied Sciences and Arts of Western Switzerland, Fribourg, Switzerland
Name and contact information for the trial sponsor {5b}	Institute of Psychology, University of Lausanne, Quartier Mouline, Bâtiment Géopolis 5138, CH-1015 Lausanne, Switzerland, stephanie.baggio@unil.ch
Role of sponsor {5c}	The sponsor/funder has no role in the study design, data collection, management, analysis, and interpretation, writing, and publication of the report

## Introduction

### Background and rationale {6a}

Adolescence, the transition between childhood and adulthood, involves many biological, psychological, and social changes, as well as identity development. It is a crucial period for health behaviours [1] and psychiatric disorders often begin in adolescence [2–4]. Adolescent mental health is therefore increasingly recognized as a public health priority worldwide [1, 5–9]. More specifically, suicide is one of the leading causes of death among young people [10]. Because it is preventable, suicide prevention has become a sustainable development goal in several countries [11, 12]. In addition, many young people who experience suicidal behaviour do not seek professional help [13, 14]. It is therefore important to reach as many young people as possible with primary suicide prevention.

Prevention strategies are usually classified either by the stage of suicidal behaviour (i.e. ‘when’) or by the recipient of the intervention (i.e. ‘who’) [15–17]. On the ‘when’ side, primary prevention occurs before there is any evidence of suicidal behaviour, secondary prevention at early stages (e.g. suicidal ideation) to detect, prevent, and treat, and tertiary prevention after suicidal behaviour has occurred to prevent disability and reduce relapses. Regarding ‘who’ is targeted in suicide prevention research, universal prevention targets the whole population regardless of risk factors, selective prevention targets subgroups at risk of suicidal behaviours, and indicated prevention targets high-risk individuals who have already experienced suicidal behaviour.

Two recent meta-analyses have summarized the evidence on effective strategies for the effectiveness of suicide prevention programmes in adolescents [16, 18]. Overall, the results are encouraging, with suicide prevention interventions reducing suicidal behaviours and psychological distress and increasing suicide awareness and helping skills. However, there are some important research gaps. One of them is the lack of studies conducted among vulnerable populations [16], who are at increased risk of suicidal thoughts and behaviours due to risk factors. Vulnerable adolescents are usually included in selective and indicative prevention, but they may also benefit from interventions such as psychoeducation (suicide awareness) used in primary prevention. This study will test the hypothesis that psychoeducation, which has already demonstrated positive effects on suicidal thoughts and psychological distress in secondary school students, is also effective for vulnerable populations [19]. This is consistent with previous calls to integrate primary and secondary prevention [13] and to include vulnerable youth in suicide prevention research [20].

### Objectives {7}

This study will evaluate the efficacy and safety of a brief primary suicide prevention intervention (i.e. psychoeducation) in vulnerable adolescents.

- O1: The primary objective is to compare the 1-week efficacy of a brief primary suicide prevention intervention in vulnerable adolescents on suicide awareness.

The study includes seven secondary objectives:

- O2: The study will test the 3-month efficacy of a brief primary suicide prevention intervention in vulnerable adolescents on suicide awareness.
- O3: The study will test the 1-week and 3-month efficacy of a brief primary suicide prevention intervention in vulnerable adolescents on knowledge of suicide.
- O4: The study will test the 1-week and 3-month efficacy of a brief primary suicide prevention intervention in vulnerable adolescents on knowledge of local resources.
- O5: The study will test the 1-week and 3-month efficacy of a brief primary suicide prevention intervention in vulnerable adolescents on suicidal thoughts and behaviours.
- O6: The study will test the 1-week and 3-month efficacy of a brief primary suicide prevention intervention in vulnerable adolescents on psychological distress.
- O7: The study will explore the proportion of access to primary suicide prevention at baseline in vulnerable adolescents.
- O8: The study will explore non-response bias.

The study also includes a safety objective, exploring the acceptability and safety (adverse events (AEs) and serious adverse events (SAEs)) of the intervention in vulnerable adolescents.

### Trial design {8}

This is a multi-centre superiority cluster-randomized controlled trial with two parallel arms. Randomization will be 1:1 with stratification on the study sites. Participants will not be blinded regarding their group attribution. The study will include a baseline assessment (*T*_0_), 1-week (*T*_1_), and 3-month (*T*_2_) follow-ups.

## Methods: participants, interventions and outcomes

### Study setting {9}

The study will take place in the French-speaking part of Switzerland. Vulnerable adolescents belonging to the following groups will be recruited for the study: (1) detained in juvenile detention centres, (2) disconnected from school, (3) in treatment for psychiatric disorders, (4) belonging to a sexual or gender diversity, (5) affected by chronic conditions, and (6) with relational/familial problems. The study includes both clinical and community sites (see study sites in Supplementary file 1).

### Eligibility criteria {10}

*Inclusion criteria* are (1) being aged 14 to 25 years old, (2) having good knowledge of French, and (3) signing the informed consent. Exclusion criteria are (1) the medical/educational staff considers that the intervention could interfere with appropriate management of an acute psychiatric disorder or may cause harm to oneself or others and (2) participants are enroled in another suicide prevention intervention during the study period. There is no restriction on the type of therapy or treatment that participants receive.

### Who will take informed consent? {26a}

The adolescent will receive an informed consent form at least 1 week before the intervention. Eligible participants will watch an information video (~5 min in length) that explains the nature of the study, its purpose, the procedures involved, the expected duration, the potential risks and benefits, any inconvenience, the voluntary nature of participation, and the possibility of withdrawing from the study at any time without consequence. The audiovisual material was created by a science filmmaker who successfully developed similar audiovisual material for a previous study [21]. The team member will then answer questions. Adolescents will sign an individual written consent form. Eligible participants who decline to participate will be free to leave or stay and participate in the workshop. The informed consent material can be found in Supplementary File 2.

In this study, we will not seek parental informed consent. Indeed, eligible participants may refuse to participate if their parents need to be involved (e.g. if parents are unaware that eligible participants are engaged in sexual and gender diversity associations) or if parents are not easily available (e.g. in detention centres). Seeking parental consent would lead to excluding the most vulnerable adolescents. However, we will encourage parental information by providing an information sheet for them and offering to answer any questions parents or legal representatives may have. In Switzerland, adolescents can consent to participate in research without the need of parental consent at 14.

### Additional consent provisions for collection and use of participant data and biological specimens {26b}

An additional consent form will be proposed to study participants for the reuse of their data for further research.

### Interventions

#### Explanation for the choice of comparators {6b}

We selected an active control, which matches the experimental intervention in terms of duration, group size, and interactive format. It will help control for non-specific effects such as group participation and attention. The active control was also chosen to avoid potential contamination of the suicide prevention messaging while still providing a meaningful experience for participants, without addressing mental health or suicide.

#### Intervention description {11a}

The framework of the primary suicide prevention intervention under study is psychoeducation. It aims to raise awareness of suicide and provide resources for seeking help for oneself and others [19]. It is delivered by Stop Suicide (www.stopsuicide.ch) and is aimed at young people aged 14 and over. The intervention lasts 90 min and is delivered in groups of 5 to 15 (average 10) participants, with a maximum of 20 to allow for fruitful interaction with all participants. The intervention provides general information about suicidal thoughts and behaviours, information about facts and myths about suicide, identification of risk factors and warning signs of suicidal behaviour, and guidance on how to get help and support for oneself and others within their community. The intervention follows a standardized format, including a lecture, group discussions based on case studies, and a quiz on facts and myths about suicide. The intervention is delivered by a trained Stop Suicide worker supported by a psychologist trained in suicide prevention for children and adolescents. The psychologist is available during and after the intervention. Despite its ‘one size fits all’ approach, the way the intervention is delivered is adapted to the group size, the setting, the age, and the socio-educational level of the young people (e.g. need for language simplification, use of shortened written case examples). A ‘resource person’ from the local team is appointed as the contact person for the participants and Stop Suicide. The resource person is available during and after the intervention on request. Additionally, participants receive a flyer with resources to contact outside their institution in case of psychological distress or suicidal thoughts.

Participants in the control group will receive a non-suicide-related intervention to improve standardization. The control intervention will be an arts-based activity, a workshop on slam poetry. It will be run by the association Slameur.ch, which already offers workshops for vulnerable groups. The duration of the control intervention and number of participants will match those of the intervention group: 90 min in groups of 5 to 15 (average 10) participants, with a maximum of 20 participants. At the end of the intervention, we will give the participants in the control group the same flyer with local resources to contact for suicide prevention. They will therefore receive a low-threshold intervention.

#### Criteria for discontinuation or modification of assigned interventions {11b}

Participants who withdraw from the study will not be replaced. They are free to discontinue participation at any time and for any reason. Participants are considered to have withdrawn if they leave the study before completion or are lost to both follow-ups. If the study team has no news of a participant, we will try to contact them to find out the reason for dropping out. If a participant withdraws due to an AE, the study team will try to collect information. The sample size calculation takes into account attrition (20%). All participants will be analysed in their original study group (i.e. intention-to-treat analyses), and all available data up to the discontinuation will be used.

#### Strategies to improve adherence to interventions {11c}

To improve study retention and encourage participation [22], we will use compensation and train the study team to search for and contact participants who have dropped out [23]. Participants who do not attend the follow-up assessment will be contacted to complete it. To avoid attrition, we plan to have a short-term primary outcome. Short-term follow-up of vulnerable populations has been described as feasible, so a 1-week follow-up is reasonable [24]. Attrition at the cluster level is not expected. For the secondary outcome at 3 months, we will contact participants by telephone. To avoid attrition, we will ask for contact details (e.g. phone number, email, postal address, phone number of a parent or caregiver). We will ask participants for their preferred means of communication and offer the use of social media (e.g. Snapchat, TikTok).

The interviewers will encourage participants to answer all missing questions on self-report instruments. The analysis strategy will help to deal with missing data by integrating multiple imputation methods.

To avoid contamination (i.e. the intervention group sharing suicide prevention information with the control group), we will plan to assess the groups at different time points. As in most settings, adolescents do not remain for extended periods; the risk of contamination bias is low. Routine care may vary between study sites. It will be adequately recorded and described for each site and included as a covariate in the models. Standardized staff training on suicidal behaviour management and suicide prevention will improve the comparability of responses to suicidal behaviour between sites. The potential effect on participants will be the same in both groups (intervention and control), so there will be no bias in conclusions about the benefits of the intervention.

#### Relevant concomitant care permitted or prohibited during the trial {11d}

Not applicable.

#### Provisions for post-trial care {30}

In the context of the ESPAIR trial, no post-trial provisions are planned. Participants will continue to have access to usual care and services provided by their respective site staff.

### Outcomes {12}

#### Nine-item perceived suicide awareness scale (PSAS-9) [25] (O1–O2)

We will use a composite measure of suicide awareness, one of the most recurring outcomes for suicide prevention programmes [19]. The PSAS-9 comprises perceived knowledge about suicide, confidence, and willingness to talk about suicide and seek help [25]. This measure is distinct from objective knowledge about suicide, which is often used as an outcome for suicide prevention programme evaluation and a proxy of suicide literacy [25]. Suicide awareness focuses on self-efficacy, a different dimension of suicide awareness that concentrates on one individual’s confidence in their ability to adopt a behaviour according to social cognitive theory [26]. The PSAS-9 was developed using a questionnaire from a suicide prevention study [25] and has already been successfully validated and used in secondary schools in Switzerland [19, 27]. Psychometric properties were acceptable (Cronbach alpha = 0.78, correlation for test-retest = 0.68, factor analyses suggesting the suitability of a one-factor model). Items are scored on a five-point scale (ranging from 0 = strongly disagree to 4 = strongly agree). A sum score of the nine questions (range 0–36) will be calculated. The final score at *T*_1_ will be used as the primary outcome (O1), while the final score at *T*_2_ will be a secondary outcome (O2).

#### Literacy of suicide scale (LOSS) [28] (O3)

The LOSS tests knowledge about suicide and its risks using 12 questions with true/false responses. The LOSS is often successfully used in suicide prevention research [29] and available in French [28]. We will compute a total score of correct responses (range 0–12). Knowledge on suicide will be assessed at *T*_0_, *T*_1_, and *T*_2_. The final scores at *T*_1_ and *T*_2_ will be used as secondary outcomes.

#### Knowledge of local resources (O4)

A questionnaire on knowledge about local resources has been developed for the project’s purpose. Five questions measured on a five-point scale (ranging from 0 = strongly disagree to 4 = strongly agree) will be used to assess whether adolescents are aware of locally available resources when they need help with suicidal behaviour. A sum score of true responses (range 0–20) will be computed. Knowledge of local resources will be assessed at *T*_0_, *T*_1_, and *T*_2_. The final scores at *T*_1_ and *T*_2_ will be used as secondary outcomes.

#### Suicidal ideation attributes scale (SIDAS) [30, 31] (O5)

This five-item measure, scored on a 0–10 scale, is designed to identify the presence and severity of suicidal thoughts. It has excellent psychometric properties and is available in French [30, 31]. A sum score ranging from 0 to 50 is computed. Suicidal ideation will be assessed at *T*_0_, *T*_1_, and *T*_2_. The final scores at *T*_1_ and *T*_2_ will be used as secondary outcomes.

#### Kessler psychological distress scale (K-6) [32, 33] (O6)

The K-6 estimates global psychological distress over the previous 4 weeks [32, 33]. This six-item measure is assessed on a five-point scale (range 0–4) and has strong psychometric properties. A French version is available. A sum score ranging from 0 to 24 is computed. Psychological distress will be assessed at *T*_0_, *T*_1_, and *T*_2_. The final scores at *T*_1_ and *T*_2_ will be used as secondary outcomes.

#### Access to primary suicide prevention (O7)

Previous access to primary suicide prevention will be assessed using three questions derived from an earlier study [34] and developed with the support of the Swiss Centre of Expertise in the Social Sciences. Participants will be asked whether they have received primary suicide prevention training in the past, including workshops, low-threshold prevention (e.g. videos, flyers), and whether they have sought information on their own (e.g. Internet, social network, family or peers). Each question will be coded as a categorical variable with three response options (‘Yes, within the past 12 months’; ‘Yes, more than 12 months ago’; ‘No’) and reported as a proportion of participants in each category. Access to primary suicide prevention will be assessed at T_0._

#### Attrition (O8)

Attrition will be coded as 1 if participants drop out of the study (drop out after *T*_0_ measure or *T*_0_ and *T*_1_ measures) and 0 if they remain in the study until the end. Attrition will be assessed as a proportion per group after *T*_2_.

#### Safety outcomes

We will systematically record SAEs, including suicide attempts and deaths by suicide before the participants leave the study site and up to 1-month post-baseline. According to the Swiss legislation, a SAE is any adverse event that [1] results in death or is life-threatening, (2) requires inpatient hospitalization or prolongation of existing hospitalization, (3) results in persistent or significant disability or incapacity, or (4) results in a congenital anomaly or birth defect. An in-depth clinical assessment by psychiatrists with an expertise in suicidal behaviours will be conducted in case of suicide attempts or deaths by suicide to rule out or establish a possible link to the intervention. Other AEs include self-harming, insomnia, appetite problems, substance use, and para-suicidal endangerment behaviours, as identified and reported by the local team before the participants leave the study site and up to 1-month post-baseline. Proportions of participants with the respective (S)AEs will be reported. At T_1_, we will also record self-reported distress due to the intervention using two questions related to the distressing and pleasurable nature of the intervention on a scale from 0 to 4 [19, 25]. We will use a sum score at *T*_1_ as the safety outcome.

In addition, we will collect the relevant following characteristics at T_0_:

#### Sociodemographic variables

We will register age (in years), parental level of education using mother and father’s job, region of origin (Swiss cantons and foreign countries), and migration status (type of residence permit). We will also ask participants whether they belong to a gender or sexual minority. We will use the Gender-Role Identity Scale (GRIS) [35], which has been validated in French. The GRIS uses 11 questions and covers three dimensions: sex assigned at birth and gender identity (questions 1 to 4, categorical items, will be reported as proportions), sexual orientation (item 5, will be reported as proportion), and gender roles and identity (questions 6 to 11, items 6 to 8 capturing masculinity and 9 to 11 femininity, combined into a single score ranging from − 2 (very feminine) to + 2 (very masculine), will be reported as mean scores) [35].

#### Educational trajectory

We will register participants’ highest level of educational attainment (type of school and grade), whether they dropped out of school, met serious school difficulties (not having the grades to pass the year, high number of absences (more than 100 periods per year), suspension days, accumulation of detention hours beyond what could be reasonably served during the academic year), had any stay in foster shelter or foster family, or had any stay in juvenile detention centres. These variables will be coded categorically, summarized as proportions.

#### Psychological and health-related variables

We will assess health literacy using the Health Literacy for School Aged Children (HLSAC-5) [36], which includes 5 questions assessed on a 4-point scale and has been validated in French [37]. A sum score ranging from 0 to 20 will be computed, and final score at *T*_0_, *T*_1_, and *T*_2_ will be used. We will ask the participants whether they have a diagnosis of a psychiatric disorder and whether they are currently taking medication for psychiatric problems. We will also ask the participants whether they have a diagnosis of a chronic condition. These variables will be coded categorically (yes/no). Adverse childhood experiences will be assessed with the Childhood Trauma Questionnaire Short Form (CTQ-SF), which includes 25 questions assessed on a 5-point scale [38]. A sum score (range 0–100) will be computed, and the final score at *T*_0_ will be used. It is well accepted by adolescents aged 14 or more [38] and has been validated in French [39]. We will also ask one question about the history of previous suicidal behaviours.

#### Study site information

Finally, we will collect the following information on study sites: canton, type of vulnerable population, clinical or community site, suicide-related routine care, and previous suicide prevention involvement. We will derive different categories for suicide-related routine care. The categorization will be developed by at least two project partners who are experts in suicide prevention. The interrater reliability will be tested with a kappa. The categorization will be trained until a kappa ≥ 0.7 is reached.

### Participant timeline {13}

Participants will be assessed at three time points: baseline (*T*_0_), 1 week post-baseline (*T*_1_), and 3 months post-baseline (*T*_2_). Participants will answer paper-based self-reported questionnaires at *T*_0_ and *T*_1_. At *T*_2_, they will answer orally during a phone call, or another preferred mean of communication. When needed (low ability to read/write), the study team will help the participant to complete the questionnaire. At T_0_, the assessment will be performed in groups, immediately followed by the intervention. At T_1_, the interviewer will approach participants at the study site. At *T*_2_, the interviewer will call or contact the participant using the selected alternative communication means. The questionnaire takes ~30 min at *T*_0_ and ~15 min at *T*_1_ and *T*_2_. To avoid loss to follow-up, the interviewer will contact participants who are unavailable at the study site for the *T*_1_ follow-up assessment to complete the questionnaire by offering alternatives such as in-person appointment or phone call.

**Fig. 1:**
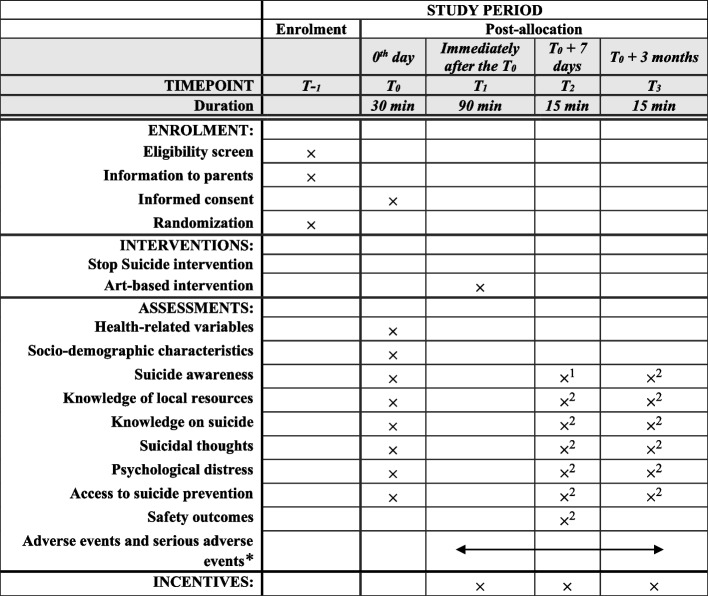
Timeline of enrolment, interventions, assessments, and incentives as per SPIRIT guidelines. ^1^Primary outcome. ^2^Secondary outcome. *Max. 1-month post-*T*_0_. The Standard Protocol Items: Recommendations for Interventional Trials (SPIRIT) Guidelines can be found at https://spirit-statement.org/publications-downloads/

Participants will receive financial compensation for their participation (a total of CHF 80—vouchers). They will receive CHF 20—after the baseline assessment, CHF 20—after the 1-week assessment, and CHF 40—after the 3-month assessment. Figure 1 provides details on the schedule of enrolment, intervention, assessments, and incentives.

### Sample size {14}

Estimates for the sample size calculation (means, standard deviations, intraclass correlation, and effect size) are based on the results of the previous trial conducted with the same intervention in adolescents in Swiss secondary schools [19]. To estimate the effect size, we used data from the intervention group (*n* = 201 who participated at baseline and follow-up). This choice was driven by the significantly higher scores at baseline in the control group compared to the intervention group. The scores of the control group remained stable over time (mean = 23 at baseline and follow-up). The scores of the intervention group were lower at baseline but increased over time (mean = 21 at baseline and 24 at follow-up, sd = 6). This three-point difference on the 0–36 point scale was used as the effect size. The expected cluster size and cluster variability are based on the expected group sizes at the study sites (from 5 to 15 participants, with an average of 10 participants). With alpha = 0.05, power = 0.80, a two-sided test, an expected cluster size *k* = 10, variation coefficient (variation in the cluster size) = 0.5 (5 to 15 adolescents per cluster), ratio cluster = 1:1, intraclass correlation = 0.05, mean = 21 in the intervention group (standard deviation = 6), and mean = 24 in the control group (standard deviation = 6), we need *k* = 20 and *n* = 200. To account for attrition, we increased the sample size by 20% (*k* = 24, *n* = 240). Even though the study is a superiority RCT, we used a two-sided test of the difference between groups, to provide a conservative sample size, in line with the recommendations of Good Clinical Practice (GCP). The sample size was computed with Stata 18 using a test to compare two independent means in a cluster-randomized design.

### Recruitment {15}

The project will run for 4 years, from October 2024 to September 2028. Data collection will take place between January 2025 and June 2027.

### Assignment of interventions: allocation

#### Sequence generation {16a}

Participants will be assigned to a group, and the group will be randomized to an intervention or control condition. Clusters will be randomized, using a 1:1 randomization with a stratification on the study sites. A random computer-generated allocation will be used in each study site, with block = 2.

#### Concealment mechanism {16b}

Clusters will be randomized using sequentially numbered, opaque, sealed envelopes. They will be opened once the creation of a group is confirmed.

#### Implementation {16c}

The Clinical Trial Unit of the Faculty of Medicine at the University of Bern will create confidential randomization lists for each site.

#### Who will be blinded {17a}

This is an open-label trial. The participants and the study team will not be blinded to their study group, as the intervention consists of scheduled workshops whose organization requires direct coordination with the study team.

#### Procedure for unblinding if needed {17b}

Unblinding will not occur, as it is an open-label study.

### Data collection and management

#### Plans for assessment and collection of outcomes {18a}

A detailed description of the instruments, including measures of validity and reliability, is provided in the ‘Outcomes {12}’ subsection. Data will be collected on paper forms at baseline and *T*_1_. Data will be entered directly into REDCap® at *T*_2_ and at *T*_1_ if this visit occurs over the phone. Data collection and extraction from the REDCap® electronic database will be carried out by trained team members. Central data monitoring will be performed through programmed checks. All source documents, including demographic data, randomization number, informed consent forms, visit dates, paper questionnaires, adverse and serious adverse events reports, will be stored in a closed cupboard at the Sponsor-Investigator’s office at the University of Lausanne for the duration of the project.

#### Plans to promote participant retention and complete follow-up {18b}

See the ‘Criteria for discontinuation or modification of assigned interventions {11b}’ and ‘Strategies to improve adherence to interventions {11c}’ subsections.

#### Data management {19}

REDCap®, a GCP-compliant online data management system, will be used to manage the collected data. This system allows the differentiation of each study team member and their function by providing personal identification for each user. Other functionalities include data security, monitoring, and automated reporting. The data will be managed and monitored by the interviewers under the supervision of Prof. Katia Iglesias, an expert in clinical trials, who will be responsible for central data monitoring for the study duration. Monitoring visits will be organized by the Clinical Trial Unit of the University of Bern.

All study data are archived for 10 years after study termination or premature termination of the study. During the study, the identification lists will be stored on the servers of the University of Lausanne. After completion of the data collection, the identification lists will be securely sent to the centres (CHUV, HUG, and Stop Suicide) for archiving on secure servers for 10 years and will be permanently deleted from the server of the University of Lausanne. Further use of the study data is detailed in the ‘Confidentiality {27}’ subsection.

#### Confidentiality {27}

Direct access to source documents will be permitted for purposes of monitoring, audits, and inspections. A restricted number of persons (investigators and interviewers) will have access to the participants’ personal details, using unique and persistent identifiers. To ensure confidentiality, this electronic list will be kept in a separate file protected by a password.

Data will be stored on the secure servers at the University of Lausanne during the project. They will be shared with the research team via electronic files (.csv files). Data will be publicly available (although upon request). Only human subject data properly de-identified and prepared under applicable legal and ethical guidelines will be entered into the online database. Once the project is completed, data (dataset and documentation) will be archived and stored in an online repository (SwissDatabase). Data will be available on SWISSUbase, and its access will require approval from the Sponsor-Investigator. Identification lists will be available at the study site for 10 years.

#### Plans for collection, laboratory evaluation and storage of biological specimens for genetic or molecular analysis in this trial/future use {33}

No biological specimens will be collected.

## Statistical methods

### Statistical methods for primary and secondary outcomes {20a}

For all statistical analyses, we will use a two-sided *α* = 0.05. We will use a Bonferroni-Holms adjustment in case of multiple testing. As participants are clustered in groups, we will use mixed-effect models, with participants nested in clusters. Statistical software will include Stata 18 and R version 4 (or new versions of these statistical software). We will use intention-to-treat analyses. We do not plan interim analyses. The use of adjusted analyses improves precision and power and will be considered as primary analyses [40]. Any deviations from the original statistical plan will be described and justified in the final trial report.

As preliminary analyses, we will examine the distributional properties of the variables, correlations, and patterns of missing data. Then, we will test whether groups (intervention vs. control) are similar at baseline using bivariable associations for all baseline characteristics and the baseline level of outcome variables. We will use standardized mean differences to compare baseline characteristics between groups, with 0.1 being considered as the threshold for balance. No inferential tests are planned for these analyses.

#### O1–O2

We will use a mixed-effect linear regression model (or negative binomial model, according to the outcome’s distribution) to test whether the primary suicide prevention intervention (intervention vs. control) predicts the score of suicide awareness (PSAS-9) at *T*_1_ (O1) and *T*_2_ (O2). We will report unadjusted and adjusted regression models. The primary analysis will be the adjusted model, controlling for baseline characteristics and using multiple imputation by chained equations (MICE) for baseline missing values on covariates [41].

#### O3–O6

For the other secondary outcomes, knowledge on suicide (LOSS), knowledge of local resources, suicidal thoughts (SIDAS), and psychological distress (K-6), we will also use mixed-effect linear regression models (or negative binomial models, according to the outcome’s distribution) with the intervention as the predictor. The same unadjusted and adjusted analyses as described above will be used. Analyses for outcomes at *T*_1_ and *T*_2_ will be performed separately.

#### O7

To explore how many vulnerable adolescents previously accessed primary suicide prevention before the study, we will compute the proportion of access to primary suicide prevention along with 95% confidence intervals. We will also explore associations between access to primary suicide prevention and participants’ baseline characteristics using bivariable and multivariable mixed-effect logistic regression models.

#### O8

To explore the characteristics of missingness and attrition, we will analyse missingness and attrition by comparing the characteristics of respondents and missing/dropouts. We will use mixed-effect linear, negative binomial, and logistic regression models, depending on the variable’s distribution. We will also discuss effect sizes using relative bias [42].

#### Safety

We will calculate the percentage of SAEs, attempted suicides, and deaths by suicide separately in each group, with 95% confidence intervals. As the number of events will presumably be very low, no inferential test is planned. We will also use percentages with 95% confidence intervals for AEs. We will calculate mean scores of self-reported acceptability using standardized mean differences between the intervention and control groups.

### Interim analyses {21b}

No interim analyses are planned. Data collection will end once all data have been collected in all study sites.

### Methods for additional analyses (e.g. subgroup analyses) {20b}

We will conduct the same analyses as described above (primary, secondary, and safety outcomes) stratified by vulnerable groups and by gender to explore whether the effect of the intervention differs between subgroups.

### Methods in analysis to handle protocol non-adherence and any statistical methods to handle missing data {20c}

We will first describe patterns of missing values (see the section Statistical methods for primary and secondary outcomes {20a}, subsection ‘O8’). In case of missing values at baseline, we will use MICE. Missing values on the outcomes will be handled by MICE (for intention-to-treat analyses) and inverse probability attrition weighting to correct our estimates for attrition [43, 44]. We will also use tipping point analysis under the missing not at random assumption to test the robustness of our findings [45]. The statistical analysis plan will provide details on the analytical strategy.

### Plans to give access to the full protocol, participant-level data and statistical code {31c}

All study documents, including the study protocol, de-identified participants’ data, and statistical codes, will be accessible through the SWISSUbase repository.

### Oversight and monitoring

#### Composition of the coordinating centre and trial steering committee {5d}

The sponsor is Prof. Stéphanie Baggio. The principal investigators (PIs) are Prof. Stéphanie Baggio and Prof. Katia Iglesias. Prof. Katia Iglesias is also the Central Data Monitor, while Prof. Stéphanie Baggio is the statistician. The monitoring institution is the Clinical Trial Unit at the University of Bern, Switzerland. The steering committee include co-authors of this protocol. In the event of study-related damage or injuries, the liability of the University of Lausanne provides compensation, except for claims that arise from misconduct or gross negligence.

#### Composition of the data monitoring committee, its role and reporting structure {21a}

This is a multi-centre trial that does not require a data monitoring committee, as this is a low-risk intervention.

#### Adverse event reporting and harms {22}

As the intervention has previously been shown as acceptable [19], we do not expect SAEs related to the study intervention. The potential discomfort created by the discussion of suicide will be handled by the psychologist, and information on local resources will be given to all participants. The study sites serve populations with high suicide risk. Suicide attempts and deaths by suicide may occur during the study period, as they do outside the study period in this population. The study sites have specific procedures for dealing with such events. In addition, we will train all staff at the study sites (e.g. medical, educational, prison staff). The intervention for the staff will be an awareness session (‘module de sensibilization’) of a standardized suicide prevention intervention for professionals given by suicide prevention experts (Groupe Romand Prévention Suicide, Stop Suicide, University of Lausanne) [46]. If a suicide attempt or a death by suicide occurs during the study period and up to 1-month post-baseline, contact will be made between the local professional team and the suicide prevention experts to determine which additional support is needed. Interventions include debriefing, group support, and specific postvention interventions for the youth and the professional team.

Several characteristics of the SAEs will be reported to the Sponsor-Investigator within 24 h, including time, duration, severity, and outcome of the event, action implemented, and link to the study participation. SAEs will be reported within 15 days to the Ethics Committee if it cannot be excluded that their occurrence is related to the ongoing study.

#### Frequency and plans for auditing trial conduct {23}

The Clinical Trial Unit at the University of Bern will conduct two site monitoring visits during the study period. Auditors conducting these visits, the Ethics Committee as well as cantonal authorities will have access to all documents and data from the study. In addition to these planned audits, a site initiation visit will be organized by the sponsor at the beginning of the study.

#### Plans for communicating important protocol amendments to relevant parties {25}

The study protocol can be amended by the Sponsor and PIs of the study. Substantial protocol amendments are submitted to the Ethics Committee for approval. In case of an emergency, deviations from the initial protocol are allowed to protect participants’ safety, rights, and wellbeing without prior Ethics Committee approval. These deviations will be reported to the Ethics Committee in the shortest possible time.

#### Dissemination plans {31a}

Research findings will be published in leading international peer-reviewed journals. One doctoral thesis will be conducted in the project. Students in medicine will also be involved in the project (master thesis and dissertation).

Data will be available for re-use after the publication of the main paper. A responsible person will approve new research proposals to avoid overlapping analyses/manuscripts.

## Discussion

The study will fill an existing research gap and provide evidence of the potential benefits of a brief primary suicide prevention intervention for vulnerable adolescents and young people. It will provide robust, evidence-based results to guide interventions to support adolescents and target vulnerable subgroups of adolescents, including evidence-based recommendations for clinical management and public health. A confirmed beneficial effect may influence recommendations for suicide prevention and lead to sustainable advances in primary suicide prevention. Indeed, the health care system should provide timely suicide prevention interventions and remove systematic barriers to adequate prevention in all groups, including vulnerable young people. The project will also fill research gaps in suicide prevention in Switzerland and thus provide an opportunity to improve suicide prevention [47].

The results of this trial project may have a substantial impact on suicide prevention by addressing the question of the benefits of primary suicide prevention for vulnerable adolescents. Strengths of this proposal include (1) unique collaboration between clinicians, epidemiologists, suicide prevention experts, social scientists, and education specialists and involvement of international experts; (2) use of a gold standard design; (3) use of a proven intervention; and (4) good statistical power.

Knowledge transfer will be ensured in the field of prevention and among professionals working with young people. We plan to disseminate the results to organizations involved in health promotion and prevention, as well as training for health professionals, school health promotion services, and public health centres and authorities. Knowledge transfer will include dissemination through written information and participation in workshops on adolescent suicide prevention. The involvement of international partners may also lead to the replication of the study in other countries and contribute to the formation of international networks that strengthen research, practice, and policy on suicide prevention for vulnerable adolescents.

Overall, this project aims to fill the current gap in high-quality evidence on primary suicide prevention among vulnerable young people. The findings may have significant policy implications by providing evidence-based support for decisions on primary suicide prevention and the protection of vulnerable young people. The results will provide decision-makers with the data needed to strengthen policies aimed at preventing suicide and protecting vulnerable young people. As suicide prevention organizations are involved in this project, the transferability of the project’s findings to fieldwork and clinical practice will also be facilitated.

### Trial status

This manuscript refers to version 6 of the protocol (August 19, 2025). Participant recruitment will start in January 2025 and is planned to be completed by June 2027. The study is expected to be completed by September 2028. The trial is registered on ClinicalTrials.gov (ID NCT06551038, registered on August 13, 2024). We used the SPIRIT checklist and the World Health Organization Trial Registration Data Set.

## Supplementary Information

Supplementary Material 1. Study sites.

Supplementary Material 2. Informed consent material

## Data Availability

The Case Report Form (French version) is available upon request to the corresponding author.

## Abbreviations

AE: Adverse event
ESPAIR: Evaluating primary suicide prevention in adolescents with risk factors
GCP: Good Clinical Practice
K-6: Kessler Psychological Distress scale
LOSS: Literacy of Suicide Scale
MICE: Multiple imputation by chained equations
PSAS-9: Nine-item perceived suicide awareness scale
SAE: Serious adverse event
SIDAS: Suicidal Ideation Attributes Scale
SPIRIT: The Standard Protocol Items: Recommendations for Interventional Trials
