# Supplementary material for: Evaluating primary suicide prevention in adolescents with risk factors (ESPAIR): study protocol for a cluster-randomized controlled trial
Source: Trials. 2025 Nov 24;26:537. doi: 10.1186/s13063-025-09266-y (PMC12642238; doi:10.1186/s13063-025-09266-y)
Supplement: Supplementary file 1 — Supplementary Material 1. Study sites. [file 13063_2025_9266_MOESM1_ESM.docx]

**Supplementary files**

1. Study sites

| Group | Site | Canton | Local investigator |
| --- | --- | --- | --- |
| Detention | Centre éducatif de détention et d’observation La Clairière^1^ | Geneva | Dr. Heller |
|  | Établissement de détention pour mineurs et jeunes adultes Les Léchaires^1^ | Vaud | Dr. Heller |
|  | Centre éducatif fermé de Parmont^1^ | Valais | Dr. Heller |
| Youth relational/familial support services | Foyer La Pommeraie^2^ | Vaud | Dr. Heller |
|  | Foyer de Chailly^2^ | Vaud | Dr. Heller |
|  | Foyers de Gilly : L’Escale et la Spirale^2^ | Geneva | Dr. Heller |
| Psychiatric treatment | Centre thérapeutique de jour pour adolescents^1^ | Vaud | Dr. Urben |
|  | Unité de soins psychiatriques fermée pour mineurs^1^ | Vaud | Dr. Urben |
|  | Service de psychiatrie de l’enfant et de l’adolescent^1^ | Geneva | Dr. Peregalli |
| Disconnection from school | Programme de mesures de transition (ForME/SEMO, Propulse)^2^ | Geneva | Mrs. Lambert |
|  | Programme intégration et formation professionnelle^2^ | Geneva | Mrs. Lambert |
|  | Le Repuis^2^ | Geneva | Mrs. Lambert |
|  | Service des Parcours Individualisés^2^ | Geneva | Mrs. Lambert |
|  | Ren’Fort^2^ | Vaud | Mrs. Lambert |
|  | CAP Formations^2^ | Geneva | Mrs. Lambert |
|  | Parcours A2mains^2^ | Geneva | Mrs. Lambert |
| Sexual and gender diversity | Dialogai/Totem^2^ | Geneva | Mrs. Lambert |
|  | Dialogai/Le Refuge^2^ | Geneva | Mrs. Lambert |
|  | Voqueer, Agnodice^2^ | Vaud | Mrs. Lambert |
| Chronic conditions | Service de psychiatrie de l’enfant et de l’adolescent^1^ | Geneva | Dr. Peregalli |

^1^ Clinical sites, ^2^ community sites.
