# Supplementary material for: Evaluating primary suicide prevention in adolescents with risk factors (ESPAIR): study protocol for a cluster-randomized controlled trial
Source: Trials. 2025 Nov 24;26:537. doi: 10.1186/s13063-025-09266-y (PMC12642238; doi:10.1186/s13063-025-09266-y)
Supplement: Supplementary file 2 — Supplementary Material 2. Informed consent material [file 13063_2025_9266_MOESM2_ESM.docx]

Demande de participation à une recherche médicale

1. Informed consent material

Titre de l’étude : Évaluer la prévention primaire du suicide chez les adolescent·e·s présentant des facteurs de risque

Titre simplifié : ESPAIR

Bonjour,

Nous aimerions te présenter l’étude ESPAIR et t’inviter à y participer. Avant d’utiliser une nouvelle méthode d’intervention, nous devons faire des recherches pour comprendre comment elle fonctionne. Une recherche comme celle-ci s’appelle une **étude clinique**.

Dans cette étude, nous voulons savoir quel effet les ateliers pour la prévention du suicide ont sur la compréhension, les connaissances et la communication sur le suicide ainsi que le mal-être. C’est pourquoi nous te demandons si tu veux participer.

Ta participation est volontaire. Ce **formulaire d’information** est là pour t’aider à décider. Tu peux poser toutes tes questions aux **personnes de référence** (voir ci-dessous). Si tu veux participer, signe la **déclaration de consentement** à la fin du document. En signant, tu confirmes avoir lu et compris les informations. Si quelque chose n’est pas clair, tu peux demander des précisions aux personnes de référence.

Le formulaire d’information et de consentement comprend quatre parties :

Partie 1 L’essentiel en bref
Partie 2 Informations détaillées sur l’étude
Partie 3 Protection des données et couverture d’assurance
Partie 4 Déclaration de consentement

Dans la **partie 1**, tu auras un aperçu général de l’étude. Dans la **partie 2**, nous t’expliquons en détail le déroulement et le contexte de l’étude. La **partie 3** contient les informations sur la protection des données et l’assurance. En signant le consentement à la fin du document, **partie 4**, tu confirmes que tu as compris les informations et que tu acceptes de participer.

Cette étude est organisée par l’Université de Lausanne, appelée le promoteur. Le promoteur est responsable de la gestion et du financement de l’étude.

Les **personnes de référence** pour cette étude sont :

Nom : Stéphanie Baggio

Adresse : Université de Lausanne

UNIL-Mouline, Géopolis 1015 Lausanne

Téléphone : +41 21 692 32 38

Courriel : stephanie.baggio@unil.ch

Partie 1 :
L’essentiel en bref

Vor allem ab Phase-3-Studien (IMP) oder konfirmatorischen MD-Studien.

# Pourquoi menons-nous cette étude ?

Le suicide est la première cause de décès chez les jeunes en Suisse. Il arrive souvent que les adolescent·e·x·s et les jeunes adultes pensent au suicide.

Dans cette étude, nous examinons si un atelier de prévention du suicide est efficace pour améliorer la compréhension, les connaissances et la communication sur le suicide chez les jeunes qui ont ou ont eu des difficultés scolaires sérieuses, des problèmes psychologiques, des maladies chroniques, qui sont issu·e·x·s de la diversité sexuelle et de genre, qui ont été placé·e·x·s dans des foyers, ou qui ont des problèmes relationnels/familiaux.. Tu en apprendras davantage sur l’étude dans le **chapitre 4**.

# Que devras-tu faire si tu participes ?

Ta participation durera 3 mois. Nous t’inviterons à 3 rendez-vous, dont un par téléphone. Un rendez-vous durera environ 15 minutes, sauf le premier qui durera 2 heures car il comprend un atelier. Le détail des rendez-vous est dans **le tableau du chapitre 5**.

Si tu décides de participer, tu seras dans le groupe d’intervention ou dans le groupe de contrôle. Dans le groupe d’intervention, tu participeras à un atelier de prévention du suicide avec l’association Stop Suicide. Dans le groupe de contrôle, tu participeras à un atelier de slam avec l’association Slameur.ch. Le groupe est choisi au hasard.

Tu en apprendras plus sur le déroulement et les procédures de l’étude dans le **chapitre 5**.

# Quels sont les bénéfices et les risques liés à la participation ?

## Bénéfices

Tu ne vas probablement pas obtenir de bénéfice direct en participant à cette étude. Cependant, ta participation peut aider de futur·e·x·s jeunes.

## Risques

Dans cette étude, parler et répondre à des questions sur le suicide ou le mal-être pourrait te rendre triste. Tu trouveras plus d’informations sur les risques et les contraintes dans le **chapitre 6**.

Partie 2 :
Informations détaillées sur l’étude

# Contexte scientifique

## Contexte : pourquoi menons-nous cette étude ?

Le suicide est la première cause de décès chez les jeunes en Suisse. Il arrive souvent que les adolescent·e·x·s et les jeunes adultes pensent au suicide. Pour les aider, il existe des programmes de prévention appelés « primaires ». Ils servent à informer sur les ressources d’aide, à mieux comprendre le suicide et à encourager à parler des pensées suicidaires avec les autres. L’idée de la prévention primaire est d’agir avant que les jeunes ne commencent à avoir des pensées ou des gestes suicidaires.

Il existe déjà des études sur la prévention primaire du suicide, qui ont montré que la prévention primaire améliore la compréhension, les connaissances et la communication sur le suicide. La prévention primaire permet aussi de diminuer les pensées suicidaires et le mal-être. Mais ces études se sont concentrées sur des jeunes à l’école obligatoire ou post-obligatoire. Nous ne savons pas encore si la prévention primaire du suicide peut aider les jeunes dans d’autres situations, où iels pourraient avoir plus souvent des pensées suicidaires. C’est par exemple le cas des jeunes qui ont des difficultés scolaires sérieuses, des problèmes psychologiques, des maladies chroniques, qui sont issu·e·x·s de la diversité sexuelle et de genre, qui ont été placé·e·x·s dans des foyers, ou qui ont des problèmes relationnels/familiaux. Un certain nombre de ces jeunes vont très bien, mais il nous manque des données pour savoir si la prévention primaire peut aider celles/ceux qui se sentent mal et permettre aux jeunes qui vont bien de continuer à aller bien.

Dans cette étude, nous examinons si un atelier de prévention primaire du suicide est efficace pour clarifier les idées, les connaissances et la communication sur le suicide chez les jeunes qui ont un risque plus grand d’avoir des pensées suicidaires. Cet atelier est déjà utilisé avec succès chez des jeunes qui sont à l’école obligatoire ou post-obligatoire en Suisse romande.

## Structure de l’étude : comment procédons-nous ?

Dans notre étude, les participant·e·x·s sont réparti·e·x·s au hasard dans 2 groupes. Cette méthode est importante pour obtenir des résultats fiables. Chaque groupe reçoit une méthode d’intervention différente.

- Le **groupe 1** (groupe d’intervention) participe à un atelier de prévention primaire du suicide, conduit par l’association Stop Suicide.
- Le **groupe 2** (groupe de contrôle) participe à un atelier de slam, conduit par l’association Slameur.ch.

## Réglementation de la recherche scientifique impliquant des êtres humains

Nous faisons cette étude selon les lois suisses (loi relative à la recherche sur l’être humain, lois sur la protection des données). Nous suivons aussi toutes les directives internationales reconnues. La commission d’éthique a vérifié et approuvé l’étude.

Notre étude est une étude nationale. Il y a 240 participant·e·x·s en Suisse romande dans au moins 8 centres (associations, foyers, centres de jour, etc.).

Tu trouveras également une description de cette étude sur le site Internet de l’Office fédéral de la santé publique, à l’adresse www.kofam.ch, sous le numéro d’enregistrement SNCTP 000006315.

# Déroulement de l’étude

## Que dois-tu faire si tu participes à l’étude ?

Tu as reçu cette feuille d’information car tu es peut-être intéressé·e·x à participer à cette étude. Pour que tes parents ou tuteur·ice·x·s soient au courant, nous leur avons aussi donné une feuille d’information (ou alors c’est toi qui l’as reçue pour leur donner si tu veux). C’est toi qui décides si tu veux participer. Tu peux en parler avec tes parents ou tuteur·ice·x·s et nous sommes disponibles pour répondre à vos questions.

La participation à l’étude est volontaire et dure 3 mois. Tu devras respecter le calendrier des rendez-vous (🡪 chapitre 5.2) ainsi que toutes les consignes données par l’équipe de recherche.

Tu devras informer l’équipe de recherche si tu commences à penser au suicide. Même si tu quittes l’étude, nous te demandons de continuer à l’informer (🡪 chapitres 5.3 et 5.4).

## Que se passe-t-il lors des rendez-vous ?

Si tu acceptes de participer, voici comment ça va se passer :

1. Lors du premier rendez-vous qui aura lieu en groupe, nous t’expliquerons les objectifs et le déroulement de l’étude. Si tu acceptes de participer, tu rempliras un questionnaire de 30 minutes sur ce que tu sais sur le suicide et ce que tu ferais si toi ou une personne proche y était confronté·e·x. Il y aura aussi des questions sur ton bien-être, tes soucis et sur qui tu es. Ensuite, tu participeras à l’atelier, qui dure 1h30.
2. Une semaine plus tard, tu rempliras le même questionnaire. Il sera un peu plus court et durera environ 15 minutes. Pour cela, nous viendrons à nouveau sur place.
3. Trois mois plus tard, tu rempliras le même questionnaire de 15 minutes. Nous te contacterons par téléphone ou un autre moyen que tu auras choisi.

Nous serons disponibles à n’importe quel moment si tu ressens le besoin de parler du suicide ou de comment tu te sens. Ces questions nous aident à savoir si l’intervention fonctionne. Nous répondons à tes questions à tous les rendez-vous. Voici la liste de ces rendez-vous :

| **Description** | **Rendez-vous 1** | **Rendez-vous 2** | **Rendez-vous 3** |
| --- | --- | --- | --- |
| Durée du rendez-vous | 2-3 heures | 15 minutes | 15 minutes |
| Lieu | Sur place | Sur place | Par téléphone |
| Date |  | 1 semaine plus tard | 3 mois plus tard |
| Explications sur l’étude et consentement | 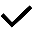 |  |  |
| Questionnaire sur qui tu es | 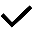 |  |  |
| Questionnaire sur le suicide | 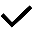 | 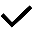 | 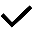 |
| Questionnaire sur le bien-être et les soucis | 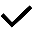 | 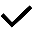 | 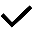 |
| Participation à l’atelier | 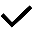 |  |  |

## Quand la participation à l’étude prend-elle fin ?

Ta participation dure 3 mois. Tu peux arrêter à tout moment avant cette date (🡪 chapitre 5.4). Tu n’as pas besoin de donner de raison. Si tu veux arrêter, dis-le aux personnes de référence ou à l’enquêtrice. Si tu arrêtes, cela n'aura pas de conséquence (🡪 chapitre 5.4).

Si tu arrêtes avant la fin de l’étude, nous te demandons de dire à l’investigatrice, à l’enquêtrice ou à la psychologue de l’étude si tu commences à penser au suicide. Même si tu arrêtes, nous pourrons encore utiliser les données que nous avons déjà. Tes données restent codées (🡪 chapitre 9).

## Que se passe-t-il si tu ne souhaites pas participer ?

Si tu ne veux pas participer à cette étude, ça ne change rien à ce que tu fais dans le centre. Tu peux assister à l’atelier si tu veux, sans avoir à répondre aux questionnaires.

# Risques, contraintes et effets indésirables

## Quels sont les risques et les contraintes liés à l’étude ?

Dans cette étude, parler et répondre à des questions sur le suicide ou le mal-être pourrait te rendre triste. Si c’est le cas, tu peux parler à la psychologue de l’étude. Elle est là pour répondre à tes questions et t’orienter pour avoir de l’aide.

# Financement et indemnisation

Cette étude est organisée par le promoteur et financée par le Fonds National Suisse pour la recherche. Les scientifiques qui y participent n’en tirent aucun avantage financier.

Si tu participes à cette étude, tu pourras choisir entre un bon-cadeau pour la Fnac ou Payot. Tu en recevras 3, après avoir rempli chaque questionnaire :

- Bon-cadeau de 20 francs après le rendez-vous 1 ;
- Bon-cadeau de 20 francs après le rendez-vous 2 ;
- Bon-cadeau de 40 francs après le rendez-vous 3.

# Résultats de l’étude

L’étude produira des résultats globaux basés sur les données de l’ensemble des participant·e·x·s (🡪 chapitre 4.1). Ces résultats ne te concernent pas directement, mais porte sur les 240 jeunes de l’étude. Si tu veux, les personnes de référence pourront te donner un résumé des résultats globaux à la fin de l’étude.

Partie 3 :
Protection des données et couverture d’assurance

# Protection des données

Nous protégeons tes données. Les lois suisses sont très strictes sur la protection des données. Tu as le droit, selon la loi suisse sur la protection des données, d'accéder à tes données, de les corriger et de les recevoir. Dans des cas exceptionnels en raison d'autres lois, ces droits ne peuvent pas toujours être garantis. Si tu as des questions à ce sujet, tu peux contacter les personnes de référence.

## Codage des données

Dans toute étude, des données sont collectées. Ces données sont enregistrées de manière codée. Le codage signifie que tes informations personnelles comme ton nom ou ta date de naissance, sont *séparées* des autres données, comme tes réponses aux questionnaires. Les informations personnelles sont conservées dans une liste dans laquelle chaque personne a un code unique. Ainsi, ton nom, ta date de naissance ou ton adresse *ne figurent pas* directement avec les autres données collectées. Par exemple, tes parents ne sauront pas ce que tu as répondu aux questionnaires. La liste d’identification est conservée à l’Université de Lausanne pendant la durée du projet, puis transférée à XXX pendant 10 ans. Personne d’autre n’y a accès, sauf exceptions (🡪 chapitre 9.5).

Lorsque nous transmettons les données de l’étude, que ce soit au promoteur ou à des spécialistes qui font des analyses, elles sont toujours codées. Cela signifie que tes données personnelles sont protégées.

## Sécurité des données pendant l’étude

Le promoteur est responsable de la sécurité de tes données. Il veille à respecter les lois en vigueur, comme celles sur la protection des données. Tes réponses aux questionnaires sont saisies sur un ordinateur et stockées sur un serveur en Suisse, à l’Université de Lausanne. Cependant, il existe toujours un risque que des personnes non autorisées puissent accéder à tes données personnelles, par exemple s’il y a un « piratage ».

## Sécurité des données après la fin de l’étude

Une fois l’étude terminée, le promoteur continue à assurer la sécurité de tes données. Selon la loi, tous les documents de l’étude, par exemple les questionnaires, doivent être conservés pendant au moins 10 ans. Après cette période, les données de l'étude sont *désidentifiées*, c’est-à-dire qu’on détruit la liste d’identification dans laquelle figure ton nom.

Les résultats de l’étude seront publiés dans des revues scientifiques. Pour cela, les données sont envoyées sous forme codée à d’autres spécialistes afin qu’iels puissent réviser la publication. Ces données ne peuvent pas être réutilisées pour d’autres projets de recherche. Une réutilisation pour d’autres projets de recherche nécessite ton consentement séparé (🡪 chapitre 9.4).

## Réutilisation et transfert de tes données pour d’autres études

Les données de cette étude sont très importantes pour la recherche future. Elles pourraient être réutilisées dans d'autres études (aussi à l'étranger).

Pour cela, un consentement séparé est nécessaire. C’est facultatif. Si tu es d’accord, tu peux lire la déclaration de consentement supplémentaire à la fin du document et la signer si tu acceptes que les données collectées pour cette étude soient réutilisées dans de futures recherches. Tu peux participer à l’étude même si tu ne signes pas ce second document.

## Droit de consultation lors des contrôles

Cette étude peut être contrôlée pour s’assurer de sa qualité. Ces contrôles sont effectués par des autorités comme la commission d’éthique. Le promoteur fait aussi des vérifications. Pour ces contrôles, un petit nombre de personnes formées a accès à tes données personnelles. Dans ce cas, les données ne sont *pas* codées. Ces personnes sont soumises au secret professionnel.

En tant que participant·e·x, tu as toujours le droit de consulter tes données.

# Couverture d’assurance

Tu seras couvert·e·x par une assurance si tu subis un dommage à cause de l’étude. La procédure est réglée par la loi. Si tu penses avoir subi un dommage à cause de l’étude, adresse-toi aux personnes de référence.

Partie 4 :
Déclarations de consentement

*La partie 4 est à lire pour ton information, elle sera signée le jour de l’atelier si tu participes à l’étude.*

Cette déclaration de consentement se compose de deux parties :

- Déclaration de consentement pour la participation à l’étude ESPAIR
- Déclaration de consentement pour la réutilisation et le transfert sous forme codée des données de cette étude pour des recherches ultérieures

Merci de lire attentivement ce formulaire. N’hésite pas à nous poser des questions si tu ne comprends pas quelque chose ou si tu souhaites des précisions. Ton consentement écrit est nécessaire pour participer.

### Déclaration de consentement pour la participation à l’étude ESPAIR

| **Numéro BASEC** | 2024-01208 |
| --- | --- |
| **Titre de l’étude** | Évaluer la prévention primaire du suicide chez les adolescent·e·x·s présentant des facteurs de risque |
| **Titre simplifié** | ESPAIR |
| **Institution responsable** (promoteur et adresse) | Université de Lausanne, Institut de Psychology, Quartier Mouline, Bâtiment Géopolis, 1015 Lausanne |
| **Lieu de réalisation** |  |
| **Investigatrice** | Prof. Stéphanie Baggio |
| **Participant·e·x** Nom et prénom en caractères d’imprimerie : Date de naissance : |  |

J’ai reçu des informations orales et écrites sur l’étude de la part de l’investigatrice ou de la personne déléguée.

L’investigatrice ou la personne déléguée m’a expliqué le but, le déroulement et les risques de l’étude.

Je participe volontairement à l’étude.

J’ai eu suffisamment de temps pour prendre ma décision. Je conserve les informations écrites et je reçois une copie de ma déclaration de consentement écrite.

Je peux mettre fin à ma participation à tout moment, je n’ai pas besoin de me justifier. Même si je me retire de l’étude, je peux bénéficier de l’aide de la psychologue de l’étude. Les données collectées jusque-là restent enregistrées et seront encore analysées dans le cadre de l’étude.

L’investigatrice peut m’exclure de l’étude à tout moment dans l’intérêt de ma santé.

J’ai compris que mes données ne sont transmises pour cette étude que sous forme codée. Le promoteur veille au respect de la protection des données conformément aux normes suisses.

Les spécialistes compétents du promoteur et de la commission d’éthique peuvent consulter mes données non codées à des fins de contrôle. Toutes ces personnes sont soumises au secret professionnel.

L’assurance responsabilité civile de l’Université de Lausanne couvre les dommages éventuels.

| Lieu, date | Nom et prénom du/de la participant·e·x en caractères d’imprimerie  Signature du/de la participant·e·x |
| --- | --- |

**Attestation** de l’investigatrice **:** Par la présente, j’atteste avoir expliqué au/à la participant·e·x la nature, l’importance et la portée de l’étude. Je déclare satisfaire à toutes les obligations en relation avec cette étude selon le droit suisse. Si je devais prendre connaissance, au cours de l’étude, d’éléments susceptibles d’influer sur la disposition du/de la participant·e·x à prendre part à l’étude, je m’engage à l’en informer immédiatement.

| Lieu, date | Nom et prénom de la personne habilitée à signer en caractères d’imprimerie  Signature |
| --- | --- |

### **Déclaration de consentement pour la réutilisation et/ou transfert de données sous forme codée**

Ce consentement ne porte pas sur ta participation à ESPAIR (🡪 chapitre 9.4).

La « réutilisation » signifie que tes données peuvent être gardées après l’étude et utilisées sous forme codée pour d’autres études.

« Transfert » signifie que tes données peuvent être transmises à d'autres scientifiques ou centres de recherche sous une forme codée pour d'autres études. Ces autres scientifiques ou centres peuvent se trouver à l'étranger. Le promoteur doit s'assurer que ce pays protège bien tes données, comme en Suisse.

| **Numéro BASEC** | 2024-01208 |
| --- | --- |
| **Titre de l’étude** | Évaluer la prévention primaire du suicide chez les adolescent·e·x·s présentant des facteurs de risque |
| **Titre simplifié** | ESPAIR |
| **Participant·e·x** Nom et prénom en caractères d’imprimerie : Date de naissance : |  |

- J’autorise la réutilisation et le transfert sous forme codée de mes données de cette étude à des fins de recherche (y compris à l’étranger). Elles pourront être utilisées dans de futurs projets de recherche, sans limite de temps.
- J’ai compris que les données sont codées et que la liste d’identification est gardée en sécurité.
- Les données peuvent être analysées et stockées en Suisse et à l’étranger. Les centres de recherche à l’étranger doivent suivre les mêmes règles de protection des données qu’en Suisse.
- Je décide librement si mes données codées peuvent être réutilisées et/ou transférées. Je peux changer d’avis à tout moment. Il suffit de le dire à l’investigatrice, sans avoir besoin de me justifier.
- Si je décide de mettre fin à ma participation, les données resteront codées.

| Lieu, date | Nom et prénom du/de la participant·e·x en caractères d’imprimerie  Signature du/de la participant·e·x |
| --- | --- |

**Attestation de l’investigatrice :** J’atteste avoir expliqué au/à la participant·e·x la nature, l’importance et la portée de la réutilisation et/ou transfert des données.

| Lieu, date | Nom et prénom de la personne habilitée à signer en caractères d’imprimerie  Signature |
| --- | --- |
